# Knowledge and behavior of cattle and sheep owners and herders regarding foot-and-mouth disease in Northern Algeria

**DOI:** 10.14202/vetworld.2019.1285-1290

**Published:** 2019-08-21

**Authors:** Ratiba Baazizi, Nora Mimoune, M’Hamed El Mokhefi, Muslim Raza, Amina Chahed, Tanveer Hussain

**Affiliations:** 1 Department of Clinic, National Veterinary High School, ENSV, Algiers, Algeria; 2 Department of Quantitative Methods, School of Business and Economics, University of Management and Technology, Lahore, Pakistan; 3 Department of Molecular Biology, Virtual University of Pakistan, Lahore, Pakistan

**Keywords:** cattle and sheep farmers, clinical signs, foot-and-mouth disease, knowledge and seniority, survey

## Abstract

**Background and Aim:**

Foot-and-mouth disease (FMD) has been occurring in Algeria since 2014, when an outbreak was announced in Setif, a district in the eastern region of the country. The problem was apparently resolved with the help of vaccination. However, in 2015, 2016, and 2018, FMD recurred. The veterinary authorities and media educated breeders on how to recognize the clinical signs and how to report the disease. This study aimed to evaluate the knowledge and recognition of FMD by farmers and breeders. Moreover, an assessment of the behavior of cattle and sheep owners and herders following FMD cases is examined.

**Materials and Methods:**

A cross-sectional survey was conducted from June to October 2018 to evaluate the perception of cattle and sheep owners and breeders regarding FMD in the Northern regions of Algeria, using questionnaires.

**Results:**

One hundred questionnaires were distributed; 71 were collected. Data showed that all the responders claimed to know about the disease, while more than half of the owners/herders claimed that they knew the clinical symptoms of FMD and mentioned fever, hypersalivation, lameness, and vesicles. Fewer than half (42%) (30/71) took some measures to prevent the disease, while more than half (58%) (41/71) did not take any measures in 2018. No one claimed to have reported the disease to authorities in 2018, while more than half had done so in 2014.

**Conclusion:**

It appears that experienced farmers recognized the clinical signs of FMD, while an academic background was not conclusively necessary for the identification of the clinical signs of the disease. Concerning the assessment of risk-associated behavior in the event of FMD occurrence, the responses of the breeders were not significantly different from those of risk-associated behaviors in the event of an epidemic. Farmers and breeders expressed similarity in terms of communicating the appearance of the disease in their livestock; the majority of them seemed to be aware of the importance of reporting the disease to local authorities, especially in 2014, when the disease first occurred. This behavior is encouraged by refund and technical assistance policies by the veterinary authorities, but in 2018, no disease was reported due to fear of slaughtering and economic loss.

## Introduction

Foot-and-mouth disease (FMD) is a highly contagious disease of domestic and wild cloven-hoofed animals across the world [1]. Worldwide, FMD has had a devastating economic impact and has been a major threat to trade [2-10]. It is ranked first among reportable diseases, and notification to the World Animal Health Organization (OIE) is mandatory [11]. FMD is caused by the FMD virus (FMDV) of the genus *Aphthovirus* within the family Picornaviridae [6,12,13]. FMDV is a single-stranded positive-sense RNA virus [10,13]. The virus has a high mutation rate and has been classified into seven serotypes: A, O, C, Asia-1, SAT1, SAT2, and SAT3, with numerous and constantly evolving subtypes showing a spectrum of antigenic diversity [5,7,10,14-16]. Several serotypes such as O, A, and Asia1 are endemic or cause periodic FMD outbreaks in the Middle East and North Africa [17,18]. The morbidity and mortality rates are higher in younger than adult, particularly in cattle. The adults heal after 10-15 days [11]. Clinically infected animals develop fever, loss of appetite, depression, hypersalivation, vesicles, and, later, erosions in or around the mouth and on the feet and teats [8,15]. Infected cattle are generally cured of the systemic infection within 8-15 days [19]. The animal in incubation excretes the virus shortly before the onset of the first symptoms and until after the clinical cure. The virus can persist for 30 months in the oropharynx of cattle, even longer in the buffalo, and approximately 9 months in small ruminants excluding pigs [11]. Frequent and uncontrolled movements of animals are the main cause of the spread of FMD outbreaks within a country, as well as from one country to another [11]. Algeria is a North African country (Maghreb region); it covers more than 2 M km², making it the largest African country. It is bordered on the north by the Mediterranean, on the west by Morocco, and on the east by Tunisia. It is subdivided into 48 administrative districts. The population is more than 42 million. Approximately 15% of Algerian workers are engaged in farming, but agriculture contributes less than the other sectors to the country’s Gross Domestic Product [20]. Livestock is an important sector of agriculture in Algeria and is mainly composed of sheep, goats, cattle, and camels. Livestock plays a major role in the socioeconomic development of millions of rural families [21]. Major FMD epidemics have been reported in many parts of the world; the disease is endemic in several African countries [22]. Algeria reported FMD in 2014, 2015, and 2018 [23-25].

In February 1999, FMD occurred in Algeria, serotype O circulation was confirmed, and the disease reported to OIE. After this episode, the authorities implemented vaccination against FMD, and no outbreak was announced. However, in 2014, an outbreak was reported [25] and the disease spread to several districts. Since this year, FMD was reported in 2015 and 2018 [23,24]. Several outbreaks were reported in Tunisia, Libya, and Morocco [26]. In general, as soon as hypersalivation and vesicles are observed, the breeders call the veterinarian, who declares to the veterinary authorities the suspicion of FMD cases.

In July 2014, the first outbreak occurred in the district of Setif; 2 months later, the disease had spread to 26 districts. Awareness campaigns and the media have played important roles in training breeders to recognize the disease symptoms to be able to report it as soon as the first vesicles are observed.

The aim of this study, conducted in 2018, was to determine if breeders are actually able to recognize and report FMD when it is suspected following the observation of symptoms. In addition, the questionnaire was designed to gather the farmer’s views on the measures taken during an epizootic and their attitudes toward vaccination.

## Materials and Methods

### Ethical approval

This study does not need ethical approval.

### Informed consents

Informed consent was obtained from all participants.

### Study area

The study was conducted in the areas of Northern Algeria where cattle and sheep breeding is important. Algeria is located in North Africa, with a human population greater than 42 million. The cattle population is estimated to be 2.2 million, the sheep population 28 million, and the goat population 5 million.

This study was undertaken on dairy farms. The farmers practiced mixture of herds, but sheep were kept in another livestock building. The study sites were selected based on the information gathered during a discussion with breeders and herders of cattle and small ruminants who had animals with signs similar to the FMD clinical case definition.

### Data collection

A cross-sectional survey was carried out between June and October 2018. One hundred farmers were asked to complete the questionnaire; 71 agreed to answer. Breeders of cattle and sheep answered a questionnaire. The survey included questions about number of animals in breeding, the probable number of animals involved when outbreaks happen (morbidity and mortality), measures taken when introducing new animals into the herds and knowledge of clinical signs by owners. The survey contained information about cases observed during the past epizootics. The seniority of the breeders was also considered.

### Statistical analysis

To compare the frequent observation of clinical signs of FMD by educational level of the breeders, Microsoft Excel Windows^®^ 2016 database and SPSS 22 (IBM, USA) were used to process the collected data. A Chi-square test was performed. To validate the results of the test, the *χ*^2^ power was calculated at the statistical significance level of 0.05. The difference was considered statistically significant where p≤0.05.

## Results and Discussion

### Outcomes of the breeders’ questionnaire survey

The questionnaire response rate of farmers was 71% (71/100); 30% (21/71) were from the east, while 70% (50/71) were from the center of the country.

#### General information

All the respondents were male (100%; n=71); 21 of them were uneducated and had never been to school, representing a rate of 29% (n=21); 14% (n=10) of those interviewed had between 25 and 30 years of experience. Those with 0-25 years of experience accounted for 42% (n=30) of the total. Of these, 7% (n=5) of respondents had <5 years of experience, 12.7% (n=9) had 5-10 years, 4.2% (n=3) had between 11 and 15 years, 4.2% (n=3) had between 16 and 20 years, and 14.1% (n=10) had over 20 years of experience. For respondents with no education, 4.2% (n=3) had between 11 and 15 years of experience, 4.2% (n=3) had between 16 and 20 years, and 21.1% (n=15) had between 20 and 35 years.

Respondents with a high level of education included 8.5% (n=6) of the total; they had already taken training. Thirty-two (n=32), corresponding to 45% of breeders, had mixed livestock, 35% (n=25) had only cattle on their farm, and 18% (n=13) had only sheep.

Of cattle breeders, 7% (5/71) had under 5 years of experience, 13% (n=9) had between 5 and 10 years of experience, 13% (n=9) had between 11 and 15 years of experience, 13% (n=9) had between 16 and 20 years of experience, and 35% (n=25) had over 20 years. Of breeders of cattle and sheep, 9% (5/57) had <5 years of experience, 16% (9/57) had 5-20 years of experience, and 44% (25/57) had over 20 years of experience.

#### Opinions and perceptions of sheep and cattle farmers regarding FMD

All respondents, regardless of the level of education, have come to recognize the signs of the disease. All the breeders who were asked about the disease knew FMD and recognized its signs because they had experienced breeding cases during epizootics that occurred in Algeria in 1999, in 2014 [25], in 2015 [24], and in 2018 [23] or from awareness campaigns through the media.

About 20% (14/71) of breeders did not know FMD, in almost all cases (n=13), because they were exclusively herders. Only one (1/71) of them also has cattle but has never seen signs of FMD in his animals.

The breeders did not take strict measures because they thought that their animals were safe from contamination due to vaccination. However, some authors report that encounters among animals in the cattle markets allow viral exchanges, permitting contamination [27].

**Figure-1:**
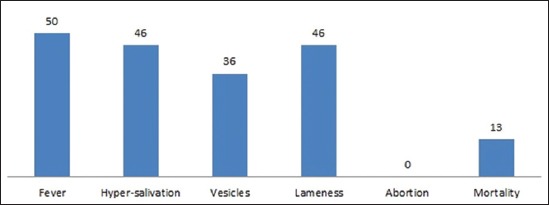
Number of breeders who have seen clinical signs.

At a general level, the fever symptoms mentioned by farmers occurred at a rate of 70% (50/71), while 65% (46/71) of the responders had seen lameness and hypersalivation, 51% (36/71) had seen vesicles, and 18% (13/71) had seen mortality. There was no (0%) abortion seen in cattle or sheep (Figure-1).

Fifty-seven interviewed breeders had cattle in their flocks, corresponding to a rate of 80% (57/71). Forty-five (45/71, 63%) of interviewed breeders had sheep on farms.

The signs mentioned by the farmers in mixed livestock included fever and vesicles with a rate of 63% (36/57), while 32% (18/57) of the responders had seen lameness or hypersalivation. No responders (0%) had seen abortion or mortality in cattle. According to sheepherders, the main clinical signs were hypersalivation and lameness at 62% (28/45), fever at 31% (14/45), and mortality at 29%, while there were no (0%) vesicles or abortion (Table-1). No breeder (0%) had observed abortion in cattle (0%) or sheep (0%), while mortality was observed only in sheep (29%), but not in cattle (0%). Concerning the sheep of breeders, 94% (30/32) of the holders with mixed breeding did not answer concerning their recognition of signs specific to FMD in sheep even if they observed symptoms as presented in the question sheet. Nearly 3% claimed to have never seen signs of the disease on sheep.

**Table 1:** Clinical signs mentioned by breeders in cattle and mixed (cattle and sheep) farms.

Signs observed by the breeders	Number of breeders who have seen signs in cattle		Number of breeders who have seen signs in sheep		*χ*^2^value (p-value)
Fever	36	63% (36/57)	14	31% (14/45)	4.07 (0.04)
Hypersalivation	18	32% (18/57)	28	62% (28/45)	3.86 (0.05)
Vesicles	36	63% (36/57)	00	0%	---
Lameness	18	32% (18/57)	28	62% (28/45)	3.86 (0.05)
Abortion	00	0%	00	0%	---
Mortality	00	0%	13	29% (13/45)	---

**Figure-2:**
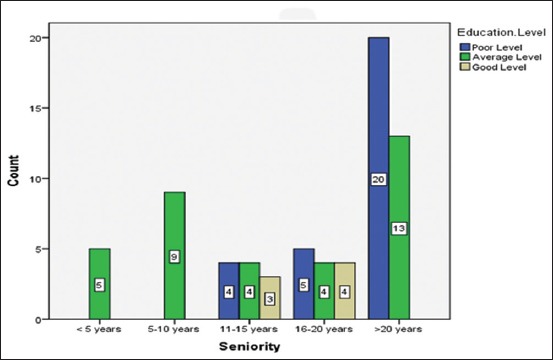
Number of respondents by years of experience.

Concerning the herders, the main clinical signs in sheep were hypersalivation and lameness (62%) (28/45), while fever occurred at a rate of 31% (14/45) and mortality was observed at a rate of 29% (13/45). Furthermore, no farmers observed vesicles (0%) (Table-1); authors have reported that signs are subtle in sheep and very difficult to diagnose in small ruminants [28]. Abortion (0%) was not seen in sheep, and authors have said that abortion is associated with FMD [4]. Sheep usually show subtle signs [28], but breeders claim that they have seen signs in sheep, which are included in the FMD statement by the OIE [24]. Breeders’ misunderstanding of the clinical signs of FMD in sheep or their confusion with signs of other more common vesicular diseases may explain the fact that they did not notice these signs because breeders know FMD as a cattle disease only. The majority of the respondents (80%, 57/71) claimed to know FMD, experience varying from 3 to 30 years (Figure-2). This is due to the media awareness campaign during the 2014 epizootic; 18% (13/71) stated that they are not concerned by FMD, as they have sheep only. Only one (1/71, 1.5%) breeder declared not to know the disease.

Of breeders who have more than 20 years of experience (1985, 33 years), a single breeder had seen signs of FMD, compared to 25 breeders with more than 20 years of experience (one breeder with 33 years and two breeders with 25 years), corresponding to an overall rate of 1/71 (14%), while the rate is 1/57 (17.5%) in one breeding case.

Of breeders with 19 years (since 1999) of experience, nine claimed to have seen the disease with a rate of 13% (9/71), while at the livestock level, the rate corresponds to 16% (9/57). Of breeders who had begun the activity in 2013, five claimed that they had seen cases of FMD, corresponding to a rate of 7% (5/71) at the global level and 9% (5/57) at the livestock level, while the disease had not been reported this year. Of breeders who began operation in 2014, nine of them claimed to have seen the disease in animals, corresponding to a rate of 16% (9/57) at the livestock level and 13% (9/71) at the global level, while 129 of animals with signs of FMD were reported by respondents. If the response rate is not high, the number of animals is important [25]. Regarding breeders with between 5 and 10 years of experience (from 2008 to 2013), nine had seen and recognized signs of FMD, corresponding to a global rate of 13% (9/71) and 19% (9/57) at the livestock level, while 40 animals showed signs of the diseases as claimed by breeders. In fact, given the seniority of the respondents, they had not experienced an epizootic since FMD was not observed between 2008 and 2013. FMD would be confused with other vesicular diseases; apparently, such breeders do not know the disease. Breeders with fewer than 5 years of experience had been performing this activity since 2013. Of these, five claimed to have seen FMD symptoms in their animals, equivalent to a global rate of 7% (5/71) and 9% (5/57) at the livestock level. These rates are low, as FMD has been reported in 2014 and 2018 [23,25], which suggests that these breeders do not recognize the signs of FMD, despite the awareness campaigns, but claim to know the disease. Regarding breeders who have claimed to have seen signs of FMD, one case was seen in 1985, but OIE was not notified. The farmers said they saw 11 cases of FMD in 1999, which is consistent with the emergence of the disease. Breeders claimed to have seen 13 cases in 2013, although FMD was not notified. These results may be due to confusion with other vesicular pathologies, as nobody had notified OIE since 1999. Regarding the year 2014, 129 cases were reported by breeders, corresponding to the recurrence of FMD following the fraudulent introduction of cattle in the district of Setif [25]. Recognition of the signs of FMD is linked to awareness campaigns conducted by the media and veterinary services, which also trained breeders to identify the disease through clinical symptoms.

In 2016, the interviewed breeders claimed to have seen FMD on 40 animals, while annual and regular vaccinations are performed. According to the declarations of the breeders, 10 vaccinated animals in 2018 showed clinical signs after their vaccination. Before 1985, not a single farmer (0%) vaccinated. However, 48% (n=34) of the farmers claimed that they had vaccinated their animals during 1999. Less than half of the respondents, 34% (n=24) said that they had not vaccinated all of their animals in the previous year (2017).

**Figure-3:**
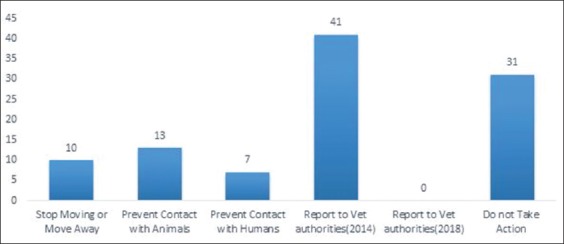
Risk factors with measures taken for foot-and-mouth disease outbreaks.

Despite the poor education of farmers, vaccination is accepted and performed in Algeria, but they believe that vaccination protects their animals from reinfection because they do not know that the vaccine is not functional in the presence of viral subtypes. Despite the level of education, the holders have taken steps to protect their animals from possible infection (Figure-3) such as stopping moving or moving away (14%) and preventing contact with other animals (18%) and with humans (10%). However, a considerable number (44%) did not take any action at all. They believe that vaccination is sufficient to protect their livestock because they are unaware that animals may be infected with other subtypes of FMDV.

Not a single (0%) report was received by veterinary authorities in 2018, while 58% reported in 2014 (Figure-3).

Furthermore, during our survey, we did not notice any disinfectant products on the farms or evidence that farmers take precautions to disinfect their boots and clothes. There was no pediluvium or rotoluve placed at the entrances of the farms. Regarding FMD’s signs after vaccination, 80% (57/71) of breeders reported having observed signs corresponding to an infection with a rate of 38% of vaccinated animals (170/448). One breeder claimed that he reported cases of FMD. Six cows were slaughtered (6/20), while the others were vaccinated, but signs reappeared 5 months later. He also claimed that there were no signs of FMD (n=240) among sheep, while small ruminants have not been vaccinated against FMD in Algeria. These observations showed that the vaccinated cattle showed clinical signs of FMD, which could be due to a vaccine failure or a break in the cold chain of the non-thermostable vaccine that requires storage between 2°C and 4°C or due to the significant mutations that the FMDV [16] causes new strains against which the vaccine used is not effective because the cross-protection between these subtypes is only partial [29].

### Statistical analysis

Concerning the clinical signs mentioned by breeders in cattle and mixed farms (Table-1), according to the results obtained, the *χ*^2^ value (p-value) shows that there was a statistically significant association between seniority and educational levels. Figure-2 represents the number of respondents differing in years of experience and their education levels. There were five respondents with <5 years of experience and average education level. For 5-10 years of experience, there were nine respondents having an average level of experience. There were four respondents with 11-15 years of experience and a poor level of education. Similarly, there were four with an average level of education and three with a high level of education with 11-15 years of experience. For >20 years of experience, there were 20 respondents having a poor education level and 13 with an average level of education. From Table-2, seniority and educational level are significantly associated (χ²=31.96, p<0.001). This signifies that there is a statistically significant association between seniority and educational levels. Cramer’s V was used to test the strength of association among groups. The strength of association between the variables (seniority and educational level) was at a moderate level (*V*=0.48). This indicates that the breeders’ experience and academic knowledge significantly influenced the occurrence of the epidemics.

**Table 2:** Chi-squared test for seniority and educational level of the surveyed breeders (n=71).

Test	Value	df	p-value
χ^2^	31.96	8	<0.001
Likelihood ratio	37.23	8	<0.001
Cramer’s V	0.48		

## Conclusion

The farmers and breeders who were interviewed claimed to recognize signs of FMD as fever, hypersalivation, lameness, and vesicles. The statistical analysis shows a significant association between seniority and educational levels. The farmers accepted vaccination, and some of them took a few measures to protect animals against the disease, while a proportion took no action. FMD is a disease that causes considerable economic losses. The disease is still circulating since 2014, despite vaccination. The breeder must recognize the disease early to avoid contagion of other animals on the farm and prevent spread to neighboring farms. Reporting by breeders must be the first link in the alert system.

## Authors’ Contributions

RB: Designed and managed this study, conducted the investigations and interviews in the field, and wrote the manuscript; NM: Collected questionnaires and wrote the manuscript; ME: Analyzed the data and wrote the manuscript; MR: Analyzed the data and wrote the manuscript; AC: The questionnaires; and TH: Analyzed the data. All the authors read and approved the final manuscript.
